# Editorial: Healthy aging, mental health, and sexuality

**DOI:** 10.3389/fruro.2023.1287189

**Published:** 2023-09-26

**Authors:** Alex Siu Wing Chan, Steve Wai Hee Chan, Elsie Yan

**Affiliations:** ^1^ Department of Applied Social Sciences, Faculty of Health and Social Sciences, The Hong Kong Polytechnic University, Hong Kong, Hong Kong SAR, China; ^2^ Urology Centre, Hong Kong Sanatorium and Hospital, Hong Kong, Hong Kong SAR, China

**Keywords:** healthy aging, mental health, sexuality, older adults, intervention, social inclusion

## Background

The global demographic landscape is currently undergoing a significant transformation, marked by an increasingly aging population. This shift serves to amplify the importance of addressing the challenges faced by older adults (1, 2). Healthy aging, a complex and multifaceted phenomenon, intricately weaves together diverse dimensions spanning physical, mental, and social well-being, as well as the often-overlooked aspect of sexuality (3). Aging-related physical changes do not inherently precipitate a decline in sexual functioning. In fact, a substantial portion of older adults maintain a desire for and actively engage in a satisfying sexual life. Research demonstrates that a significant number of men and women continue to participate in sexual activities well into their later years, with regular sexual expression correlating with sound physical and mental health. The sustenance of robust physical and mental health, a constructive attitude towards sexuality in later stages of life, and access to a compatible partner collectively contribute to the perpetuation of sexual activity (4, 5). This avenue of research serves as a poignant reminder of the intricate and interconnected factors that influence the comprehensive well-being of older adults. It underscores the profound interplay between healthy aging, mental health, and sexuality, all of which fundamentally contribute to shaping the overall quality of life for older adults. Among the contributing factors that heighten the vulnerability of older adults to mental health concerns are compromised physical health, a history of mental health disorders, significant life transitions, the onset of medical ailments, personal losses, experiences of social isolation, and adverse life events (6). These elements collectively emphasize the need for a comprehensive understanding of the challenges that older adults face, spanning multiple domains of their lives.

## The intersection of healthy aging, mental health, and sexuality

Obsa et al. delved into the risk factors of pelvic organ prolapse among women at a teaching and referral hospital, identifying several contributing factors such as age, parity, and menopausal status. Consequently, healthcare professionals were better equipped to devise prevention and treatment approaches that could substantially impact women’s sexual and holistic health throughout the aging process. In another study, Hu et al. scrutinized an evaluation method for product design solutions aimed at fostering healthy aging companionship. This research emphasized the importance of creating products that facilitated companionship and social interaction among older adults, ultimately contributing positively to their mental and emotional well-being. The study underscored the need for product designers to consider the unique needs and challenges confronted by older adults when devising products that could bolster healthy aging.

## Designing for companionship: promoting mental health in aging

Focusing on China, Wang et al. investigated loneliness, anxiety, and depressive symptoms among older adult migrants. The study discovered that perceived stress and resilience served as mediators between these psychological factors, highlighting the necessity for social and emotional support for older adult migrants. This research illuminated the importance of addressing the unique challenges faced by older adult migrants and formulating efficacious strategies to bolster their mental and emotional well-being. Cheng et al. explored the prevalence and risk factors correlated with multimorbidity, falls, and fear of falling among older adults in eastern China. Understanding these factors was indispensable in devising effective prevention and treatment approaches that could foster healthy aging and avert falls and related injuries.

## Intergenerational relationships and depressive symptoms in the older adults

Intergenerational relationships play a vital role in the emotional well-being of older adults and can significantly impact their depressive symptoms. These relationships involve interactions between individuals from different age groups, typically older adults and their children, grandchildren, or even great-grandchildren. Positive intergenerational relationships can serve as a source of emotional support, companionship, and a sense of purpose for older adults.

The relationship between the brain, aging, and depression among older adults is a complex and multifaceted one. As individuals age, their brains undergo various structural and functional changes that can influence their susceptibility to depression (7, 8).

**Figure 1:**
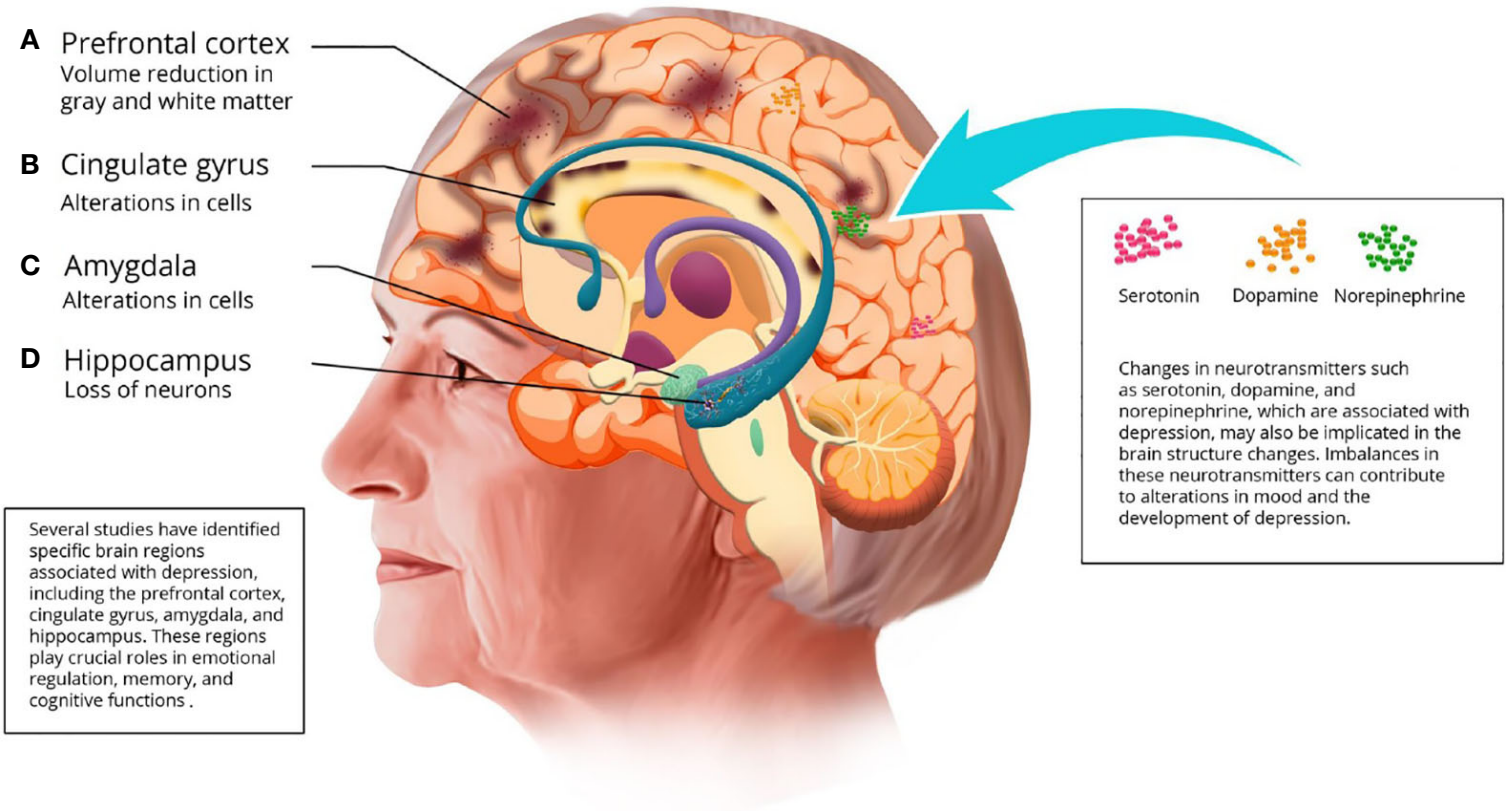
Correlation between brain structure, aging and symptoms of depression. **Figure 1** indicates that the prefrontal cortex, cingulate gyrus, amygdala, and hippocampus are interconnected brain regions that collectively play a crucial role in regulating emotions, memory, and mood. When considering their correlation with depression among aging individuals, it's important to understand how changes in these brain regions can contribute to depressive symptoms: **(A)** Prefrontal Cortex (PFC): The PFC, particularly the dorsolateral prefrontal cortex (DLPFC), is involved in executive functions, decision-making, and emotional regulation. In aging individuals, there can be a decline in PFC function, which may lead to difficulties in managing emotions and making adaptive choices. This decline can contribute to depressive symptoms, as individuals may struggle to cope with life changes and stressors. **(B)** Cingulate Gyrus: The cingulate gyrus, a part of the limbic system, is involved in emotional processing and regulation. Changes in the cingulate gyrus, such as alterations in connectivity, can affect emotional control. Dysfunction in this region has been linked to depression in older adults, as it can lead to heightened emotional responses and difficulties in regulating negative emotions. **(C)** Amygdala: The amygdala is responsible for processing emotions, particularly fear and anxiety. In older adults with depression, the amygdala may show increased activity or abnormal functioning, leading to heightened emotional reactivity and persistent negative emotional states. **(D)** Hippocampus: The hippocampus is vital for memory consolidation and spatial navigation. In aging individuals, the hippocampus can undergo structural changes, such as atrophy and decreased neurogenesis. These changes are associated with memory problems and an increased risk of depression. Additionally, a smaller hippocampus has been linked to a poorer response to treatment for depression.

One of the key factors in this relationship is the natural aging process itself. As people age, there is a gradual decline in cognitive functions, such as processing speed and working memory, which can affect how they perceive and react to life's challenges. These cognitive changes can contribute to feelings of frustration and helplessness, potentially increasing the risk of depression (9). Moreover, age-related changes in brain chemistry also play a role. Alterations in neurotransmitter systems, such as serotonin and dopamine, have been linked to mood disorders like depression. These changes can make older adults more vulnerable to developing depressive symptoms (**Figure 1**; 10).

The correlation between these brain regions and depression among aging individuals highlights the intricate neurobiology of late-life depression. Changes in brain structure and function in these regions can contribute to the onset and persistence of depressive symptoms in older adults (11). Additionally, older adults often face significant life transitions and losses, such as retirement, the death of loved ones, or declining physical health (12). These stressors can trigger or exacerbate depression, and the way the brain processes and copes with these stressors may change with age.

Zheng et al. conducted a study on CHARLS data, examining the effect of intergenerational exchange patterns and intergenerational relationship quality on depressive symptoms in the elderly. This research underscored the importance of addressing mental health concerns in older adults, especially those with chronic illnesses, by understanding the complex interrelation between mental health, age, resilience, and frailty.

Furthermore, Zhang et al. investigated the interplay between health beliefs, lifestyle, and cognitive aging among Chinese community residents. By understanding this complex interrelation, healthcare professionals could develop targeted interventions that promoted healthy aging and prevented cognitive decline. 

## Access to psychological assistance for older adults: prevalence and determinants

Meng et al. probed the interrelation between depression, age, resilience, and frailty among HIV-positive adults. This investigation emphasized the need to address mental health concerns in older adults, particularly those grappling with chronic ailments like HIV. Additionally, Coşkun et al. scrutinized the prevalence and determinants of psychological assistance services for older adults in Turkish society. This research accentuated the importance of accessible and culturally tailored mental health services for older adults.

Lastly, Cao et al. investigated the impact of hearing loss on cognitive impairment and explored the mediating role of depressive symptoms and the moderating role of social relationships. This research highlighted the importance of addressing hearing loss and its potential impact on cognitive function, as well as the need to consider the mediating and moderating factors that could influence this relationship.

## Addressing the unique needs and challenges among older adults

Psychological well-being entails maintaining positive relationships with others and living a life filled with purpose and significance. Research has shown that individuals who possess a positive psychological well-being tend to experience greater ease and derive more satisfaction from their lives (13). Understanding and addressing the unique needs and challengesof healthy aging, mental health, and sexuality to promotecomprehensive well-being in older adults.

Understanding and addressing the unique needs and challenges of healthy aging, mental health, and sexuality to promote comprehensive well-being in older adults. By comprehending these concerns and devising efficacious prevention and treatment strategies, we can facilitate healthy aging and aid older adults in maintaining a gratifying and satisfying intimate life. It is imperative to persist in investing in research and formulating policies and programs that endorse healthy aging while bolstering older adults’ mental and physical health. One of the cardinal takeaways from this Research Topic is the significance of apprehending the distinctive needs and challenges faced by older adults. Whether it involves understanding the risk factors correlated with pelvic organ prolapse or conceiving products that foster companionship and social interaction among older adults, it is vital to contemplate the specific needs and challenges encountered by older adults when developing prevention and treatment strategies.

The aging gut undergoes various transformations that encompass the mechanical breakdown of food, gastrointestinal motility, food passage, integrity of the intestinal wall, and chemical digestion (14). These modifications progressively contribute to a diminished capacity to provide the body with sufficient nutrient levels, ultimately leading to the emergence of malnutrition. Moreover, research indicates that gastrointestinal disorders, and particularly malnutrition, detrimentally impact the quality of life. A deeper comprehension of the pathophysiology underlying malnutrition in the elderly is imperative to enhance our understanding of age-related shifts in appetite, dietary intake, internal balance, and body composition. This knowledge is pivotal in formulating more effective strategies for prevention and intervention, aimed at fostering healthy aging (15). Another salient takeaway is the importance of addressing mental health issues in older adults. Depression, anxiety, and loneliness are pervasive concerns among older adults, particularly those contending with chronic illnesses. By crafting potent strategies to bolster the mental and emotional well-being of older adults, healthcare professionals can champion healthy aging and enhance the overall quality of life for older adults.

The Healthy Ageing Strategy encompasses pivotal domains that seamlessly converge to facilitate mobility, foster secure engagement, and nurture meaningful interactions. These foundational areas encompass the promotion of physical activity, the enhancement of mobility and connectivity, the assurance of adequate housing, employment, and financial stability, the prioritization of safety, the facilitation of continuous learning and knowledge exchange, and the holistic approach to health while embracing diversity. These holistic efforts collectively empower and equip individuals to lead active, engaged, and thriving lives within our vibrant community (16). For instance, the Healthy Ageing Strategy of New Zealand paints a vision where senior citizens bask in a high quality of life, embrace graceful aging, and find solace in a dignified end-of-life phase, all within communities tailored to cater to their unique needs. Similarly, the Inner West Council’s Healthy Ageing Strategy for 2022-2025 in Australia expounds upon the Council’s responsibilities, delineating strategic domains that endorse secure participation and foster enriching lifestyles among individuals (17).

## Navigating the nexus of healthy aging, mental health, and sexuality

The World Health Organization (WHO) provides an encompassing definition of sexuality that spans a wide spectrum of human experiences. It includes sex, gender identities and roles, sexual orientation, eroticism, pleasure, intimacy, and reproduction. This multifaceted aspect of life is not limited to physical actions but extends to thoughts, fantasies, desires, beliefs, attitudes, values, behaviors, practices, roles, and relationships. It’s profoundly influenced by an array of factors ranging from biological and psychological to societal, economic, political, cultural, ethical, legal, historical, religious, and spiritual dimensions (18, 19).

Within the context of adulthood, factors related to sexuality take on a profound significance. Life satisfaction, often regarded as a fundamental indicator of successful aging, is a composite of several elements, including the absence of disease, good physical and mental health, active social participation, and overall contentment with life. Yet, it’s remarkable that the sexual dimension of aging has been relatively underexplored within the broader framework of successful aging and warrants a more focused examination (20).

Sexuality is an integral component of a fulfilling adulthood. Engaging in sexual activity can exert a positive influence on emotional, mental, and physical well-being, thereby contributing to an enhanced quality of life. With the extension of human lifespans and an increasing number of older individuals engaging in sexual activity, there’s a corresponding rise in older adults aged 65 and above seeking medical guidance for various sexual concerns or challenges (21). This shifting landscape reflects evolving perspectives, beliefs, and attitudes that underscore the significance of maintaining a healthy sexual life in later stages. Furthermore, ongoing developments in medical treatments and interventions have empowered individuals to sustain a satisfying sexual life irrespective of their age, effectively facilitating continued sexual activity as people progress into their later years. As we delve into the intricate interplay between healthy aging, mental health, and sexuality in this Research Topic, it becomes evident that these facets of life are not isolated but rather deeply interconnected. This Research Topic presented in this collection shed light on various dimensions of this correlation, offering valuable insights that contribute to a comprehensive understanding of how these factors influence one another.

## Conclusion

Successful aging can be assessed from both a community and an individual perspective. From a community standpoint, it is characterized by factors related to health and active participation in strategies to enhance well-being. Conversely, for an individual, it is defined by considerations such as physical health, biological and mental function, and social engagement. As healthy aging is a complex concept encompassing various dimensions of physical, functional, cultural, and mental well-being, it is crucial to consider all these aspects when analyzing the process, both from empirical data and personal circumstances. In essence, meaningful results cannot be obtained by solely examining either the individual or the community, as multiple factors contribute to the overall health of older adults (22). 

This Research Topic underscores the importance of advocating healthy lifestyles and beliefs to encourage healthy aging and cognitive function. By fostering salubrious behaviors such as regular exercise, wholesome eating, and social interaction, healthcare professionals can advance healthy aging and support older adults’ physical and mental well-being (23). Discerning the critical issues associated with healthy aging, mental health, and sexuality is essential in promoting healthy aging and ameliorating the overall quality of life for older adults (16). By investing in research, formulating effective prevention and treatment strategies, and endorsing healthy lifestyles and beliefs, we can assist older adults in sustaining a fulfilling and satisfying intimate life and advocate healthy aging for generations to come. It is crucial to prioritize the unique needs and challenges confronted by older adults to ensure they receive the support and care requisite for living healthy, gratifying lives.

## Author contributions

AC wrote this editorial. SC and EY provided feedback on earlier drafts. All authors approved of the final submission.
